# Editorial: Editorial on multidimensional physiology: novel techniques and discoveries with bioimpedance measurements, volume II

**DOI:** 10.3389/fphys.2025.1729190

**Published:** 2025-11-07

**Authors:** Meng Dai, Lin Yang, Zhanqi Zhao, Fernando Seoane

**Affiliations:** 1 Department of Biomedical Engineering, Fourth Military Medical University, Xi’an, China; 2 Department of Aerospace Medicine, Fourth Military Medical University, Xi’an, China; 3 School of Biomedical Engineering, Guangzhou Medical University, Guangzhou, China; 4 Department of Clinical Science, Intervention and Technology, Karolinska Institutet, Stockholm, Sweden; 5 Department of Clinical Physiology, Karolinska University Hospital, Stockholm, Sweden; 6 Department of Medical Technology, Karolinska University Hospital, Stockholm, Sweden; 7 Department of Textile Technology, University of Borås, Borås, Sweden

**Keywords:** bioimpedance (BIA), electrical impedance tomography, muscle activities, body composition analysis, tissue dielectric properties, cerebral perfusion, regional lung function, ventilation/perfusion (V/Q) matching

## 1 Introduction

The field of physiological monitoring is undergoing a transformative shift, moving beyond traditional, often invasive, measurements towards integrated, dynamic, and non-invasive assessments. This paradigm change is largely driven by advancements in functional imaging and sensing technologies. Among these, electrical bioimpedance (EBI) and electrical impedance tomography (EIT) have emerged as powerful, versatile tools capable of providing unprecedented insights into the human body’s complex functions. This special issue, “Multidimensional Physiology: Novel Techniques and Discoveries with Bioimpedance Measurements, Volume II,” the expansive and innovative applications of these technologies are presented. The collected articles highlight a compelling journey from foundational tissue characterization and metabolic health assessment to real-time, bedside monitoring of organ function, underscoring the growing importance of EBI and EIT in both research and clinical settings.

## 2 Applications of electrical bioimpedance (EBI)

### 2.1 Body composition analysis for weight management

Pescari et al. extensively utilized bioelectrical impedance analysis (BIA) for dynamic body composition assessment in women with obesity. The TANITA body composition analyzer (BC-418 MA III) was employed to monitor anthropometric and bioimpedance parameters over a 12-week dietary intervention. Key metrics evaluated included body weight, basal metabolic rate, total body fat percentage, trunk fat, muscle mass, fat-free mass, and hydration status. BIA was instrumental in demonstrating the superior long-term benefits of isocaloric diets with specific macronutrient distributions (high-protein, low-carbohydrate, and ketogenic diets) in optimizing body composition and central adiposity compared to calorie-restrictive diets and time-restricted eating. For example, the high-protein diet significantly reduced fat mass (3.66% reduction compared to low-energy diet) and increased muscle mass (3.61% increase compared to LED), as revealed by BIA. Conversely, the very low-energy diet was associated with an unexpected increase in waist circumference and less pronounced fat and muscle mass reductions. This highlights BIA’s crucial role in providing detailed insights beyond simple weight or BMI changes, essential for evaluating dietary efficacy.

### 2.2 Physical activity and muscle activity analysis

Hafid et al. investigated the potential of EBI, specifically electrical impedance myography, for analyzing physical activity and human motion recognition. This application moves beyond traditional motion capture systems by offering insights into underlying muscle involvement. By analyzing EBI signals from the quadriceps muscle and extensor digitorum longus muscle during four lower body physical activities (squats, lunges, balance walk, and short jumps), the study demonstrated that each activity exhibited unique EBI signal characteristics. These distinct patterns provided information on individual muscle activation, mass involvement, coordination, and balance challenges during movement. For instance, the quadriceps muscle generally showed larger amplitudes and greater variability in features like squat/lunge position amplitude, reflecting its role as the primary engaged muscle. Fluctuations in EBI signals during a balance walk correlated with visible balance issues, suggesting muscle compensation for instability. This non-invasive method has implications for sports training, rehabilitation, and detecting age-related functional declines.

### 2.3 Foundational measurement of tissue dielectric properties

Shi et al. focused on a foundational application of EBI: the optimized measurement of dielectric properties (conductivity and permittivity) of active biological tissues across the 10Hz-100 MHz frequency range. These properties are crucial as they contain rich information about tissue morphology, structure, composition, and functional state, vital for disease diagnosis and electrical impedance imaging. The study developed a dual-purpose measuring cell and an integrated methodology combining four-electrode and two-electrode techniques to overcome challenges like electrode polarization at low frequencies and distributed parameters at high frequencies. This approach achieved high accuracy and repeatability in measuring these properties in NaCl solutions (average deviation less than 1.5%, maximum 6.34%) and porcine liver tissues (overall relative deviation below 6%). This robust tool is essential for advancing biomedical research that relies on understanding tissue dielectric behavior.

## 3 Applications of electrical impedance tomography (EIT)

### 3.1 Cerebral perfusion heterogeneity assessment

Zhu et al. applied EIT for evaluating cerebral perfusion heterogeneity, which indicates uneven blood flow in the brain, common in stroke, brain tumors, and epilepsy. EIT is presented as a non-invasive functional imaging technique that measures boundary voltages to estimate tissue electrical properties. The researchers developed and validated novel quantitative indices: the global inhomogeneity (GI) and asymmetry index (AI), as robust metrics for intracranial perfusion distribution. Using unilateral carotid artery compression, EIT accurately captured changes in cerebral perfusion heterogeneity, with GI and AI values significantly lower in the non-compressed state (P < 0.001). Receiver operating characteristic analysis confirmed their diagnostic value (area under curve for GI was 0.94, and for AI was 0.86) in detecting abnormal cerebral perfusion heterogeneity. This application offers a non-invasive, real-time, continuous bedside monitoring solution.

### 3.2 Regional lung function assessment

Sang et al. explored EIT’s use in assessing regional lung function, particularly during forced vital capacity (FVC) maneuvers. EIT is described as a radiation-free imaging technique that measures regional ventilation distribution over time through impedance changes. The study investigated how the electrode belt’s measurement plane influences the spatial and temporal distribution of regional lung function parameters. Findings indicated that the measurement plane significantly impacts parameters like FEV1_EIT_, FVC_EIT_, and FEF25%–75%_EIT_. The study recommends standardizing to the same measurement plane for intra-subject comparisons, specifically suggesting the fourth intercostal space for sitting subjects performing lung function tests, as it yielded the most homogeneous functional EIT images. This standardization is vital for accurate interpretation of EIT data in clinical diagnostics.

### 3.3 Ventilation/perfusion (V/Q) matching calibration in lungs

Han et al. advanced EIT’s clinical utility for V/Q matching assessment in the lungs, critical for managing acute respiratory complications like pulmonary embolism and atelectasis in ICU patients. To overcome the invasiveness of traditional cardiac output monitoring for V/Q ratio calculations, they proposed a novel calibration method based on arterial blood pressure (BPCM). The study validated that arterial blood pressure waveform integration (AUC × heart rate) showed a strong correlation with cardiac output (*R* ^2^ = 0.80, p < 0.001), demonstrating its utility as a surrogate for cardiac output in V/Q matching calibration. Both the proposed BPCM and the conventional cardiac output calibration method (COCM) provided enhanced V/Q match region segmentation compared to the area limited method, effectively distinguishing “low ventilation areas” and “low perfusion areas” in a piglet model. The BPCM demonstrated comparable efficacy to COCM in delineating V/Q mismatch regions, with a slightly higher correlation for the low perfusion index with the PaO_2_/FiO_2_ ratio (r = 0.49 vs. 0.44 for COCM). This less invasive approach holds significant promise for continuous bedside V/Q mismatch assessment.

## 4 Conclusion

The research presented in this collection unequivocally demonstrates that EBI and EIT have matured into indispensable modalities within the physiological monitoring toolkit. The studies span a remarkable spectrum—from optimizing dietary interventions through precise body composition analysis with BIA, to decoding muscle activity for rehabilitation, and establishing robust foundations for tissue dielectric property measurement. Furthermore, EIT has proven its clinical mettle by offering non-invasive, real-time solutions for assessing cerebral perfusion heterogeneity, regional lung function, and ventilation/perfusion matching, which are critical for managing complex ICU patients. The collective findings not only validate the accuracy and utility of these techniques but also chart a course for the future of personalized medicine. By providing detailed, dynamic, and safe physiological data, EBI and EIT are poised to revolutionize how we diagnose, monitor, and treat a wide array of conditions, ultimately paving the way for more informed clinical decisions and improved patient outcomes.

